# Editorial: Translational study for amyotrophic lateral sclerosis treatment

**DOI:** 10.3389/fneur.2022.1105360

**Published:** 2023-01-16

**Authors:** Sven Schröder, Gerhard Litscher, Weidong Pan

**Affiliations:** ^1^HanseMerkur Center for TCM at the University Medical Center Hamburg-Eppendorf, Hamburg, Germany; ^2^Research Unit of Biomedical Engineering in Anesthesia and Intensive Care Medicine, Department of Anesthesiology and Intensive Care Medicine, Medical University of Graz, Graz, Austria; ^3^Research Unit for Complementary and Integrative Laser Medicine, Department of Anesthesiology and Intensive Care Medicine, Medical University of Graz, Graz, Austria; ^4^Traditional Chinese Medicine (TCM) Research Center Graz, Department of Anesthesiology and Intensive Care Medicine, Medical University of Graz, Graz, Austria; ^5^Department of Neurology, Shuguang Hospital Affiliated to Shanghai University of Traditional Chinese Medicine, Shanghai, China; ^6^Laboratory of Brain Science, Shuguang Hospital Affiliated to Shanghai University of Traditional Chinese Medicine, Shanghai, China

**Keywords:** amyotrophic lateral sclerosis, translational research, genetic-environmental studies, integrative therapies, genotype-phenotype, Vital Capacity

Amyotrophic lateral sclerosis (ALS) is a motor neuron disease characterized by progressive loss of upper and lower motor neurons. Despite significant scientific efforts in the last decades, many aspects of ALS, including causes, prevention, diagnosis, prognostic markers, and therapy, are still not completely understood or fully adequate for patient care. Hence, translational research converting results from basic research and empirical knowledge into outcomes that directly benefit humans is necessary with a bench-to-bedside approach.

Approximately 5–10% of total ALS cases are familial (fALS), of which 20% are linked to a point mutation of Cu/Zn superoxide dismutase (SOD1) (1). SOD1 variants are reported in 15% of people with familial ALS in European populations, in 30% of people with familial ALS in Asian populations, and in 1–2% of people with apparently sporadic ALS in both populations (2). However, most fALS and sporadic cases could not confirm a genetic cause. The C9orf72 hexanucleotide repeat expansion (HRE) is associated with an accelerated deterioration of respiratory function and decreased survival (3, 4). However, few studies have investigated the association between the C9orf72 repeats length and the survival of ALS patients without C9orf72 HRE, which is primarily of significance in Chinese ALS patients who usually do not carry the C9orf72 HRE. Tang et al. discovered that the repeat length of C9orf72 is associated with the survival of ALS patients without C9orf72 pathological expansions. This fact is relevant because the length of two in the maximum C9orf72 repeat allele was identified to be associated with favorable survival in ALS patients without C9orf72 HRE. The findings are a helpful hint for basic research on the detrimental role of C9orf72 pathological repeats, for the determination of the cut-off value of C9orf72 pathological repeats, and might become relevant for genetic counseling.

The relevance of the genotype-phenotype correlation was demonstrated for autosomal dominant lower extremity-predominant spinal muscular atrophy-1 (SMALED1), another neurodegenerative disease. Although numerous cases have been reported associated with DYNC1H1 mutations, the correlation to the severity of the phenotype could not be established (5). The current study by Li, Zhu et al. reported DYNC1H1 gene c.1792C>T (p.R598C) and c.790C>G (p.R264G) *de novo* heterozygous mutations in two sporadic SMALED1 cases, of which the latter was a novel DYNC1H1 mutation. In addition, the authors found that mutations in the DYN1 region of the DYNC1H1 protein were associated with a more severe phenotype, more complications, and increased Central Nervous System (CNS) involvement compared to the DHC_N1 region. These findings expanded the mutational and clinical spectrum of dyneinopathies but also shed light on the potential of gene sequencing techniques and gene therapy.

In addition to purely genetic causes, gene-environment interaction may play a role in ALS. Yoshida reviewed genetic-environmental studies on ALS and Parkinsonism–dementia complex (PDC) in the regions of Guam, Kii, and Oshida. There was strong evidence that metallic environmental elements, such as Ca, Mg, Al, Fe, Cu, and Zn, play a relevant role in the oxidative process of neuronal degeneration. Hence, the author highlighted the importance of regulating ionic homeostasis to prevent the development and progression of neurodegenerative diseases. Finally, he also drew attention to the importance of synchrotron radiation (SR)-based studies because they provide non-destructive analyses, chemical state analyses, and imaging distribution of the elements at a single cellular level. To elaborate therapeutic strategies, SR seems crucial to elucidate the role of transition metals (i.e., Mn, V, and/or Ti) in oxidative stress on neurons.

The percent-predicted Forced Vital Capacity (FVC%) is generally used to evaluate the respiratory function in ALS. To find the optimal evaluation parameter of respiratory function in patients with bulbar ALS, Huang et al. compared the relevance of percent-predicted Slow Vital capacity (SVC%) and FVC% in a prospective study with 51 patients. It was found that the SVC% was significantly higher and more reflective of actual respiratory function than the FVC%. Furthermore, the severity of dysarthria correlated positively with SVC% minus FVC%. The results provided improved clinical markers for predicting the outcome of ALS with bulbar involvement with a tentative impact on clinical decisions.

Currently, the only established drug for ALS is riluzole, a glutamate antagonist, which causes minor side effects in some patients with ALS and, very rarely, serious adverse events. Edaravone, a free radical scavenger, is given only intravenously and is not approved in many countries. While some studies described how riluzole improves patient survival in clinical trials and population studies (6), other studies concluded that none of these drugs can ameliorate the progression of the disease in a significant manner (7, 8). Hence, there is an urgent need for new and innovative treatments for ALS.

*Mouse Nerval growth factor* (mNGF) is a biologically active protein extracted from the submaxillary gland of mice. It is officially approved in China for treating peripheral nerve injury, but no study has examined its use in ALS. Li, Dong, Qian et al. published a retrospective observational study injecting mNGF in 32 ALS patients as an add-on therapy to riluzole in comparison with 64 patients receiving riluzole only. There was no statistically significant difference between the groups regarding *ALS Functional Rating Scale* (ALSFRS-R) or decline in *body mass index*. Of the patients treated with riluzole+mNGF, 12.5% reported intermittent headaches and dizziness, and one patient experienced diarrhea. To our knowledge, this is the first report on the use of mNGF in ALS patients. Despite the negative results, it is worth conducting randomized clinical trials based on a larger sample size and longer follow-up periods to reach a final conclusion.

Another innovative research approach is the external application of traditional Chinese herbal medicine. The randomized, triple-blinded ALS-CHEPLA (Schröder et al.) study compared the efficacy and safety of applying *Ji-Wu-Li* plaster (JWLP, *n* = 60) with placebo plaster (PLAP, *n* = 60) for 20 weeks as an add-on therapy. JWLP showed clinical efficacy, as measured by the ALSFRS-R, ALS-SSIT, and weight loss. Systemic adverse events were mild, temporary, and considered unrelated to the intervention. Local allergic dermatitis occurred similarly in both groups. The herbs of JWLP targeted the pathophysiological mechanism of ALS oxidative stress, neuroinflammation, mitochondrial dysfunction, apoptosis, and skeletal muscle atrophy. Hence, JWLP may offer a promising and safe add-on therapy for ALS, but a confirmative long-term study is required.

This Research Topic illuminates new aspects of ALS regarding cause, prevention, diagnosis, prognostic markers, and therapy. Furthermore, the Research Topic shows that innovative approaches, including neurogenetics, genetic-environmental studies, optimization of diagnostics and predictive parameters, and experimental and herbal therapies, are possibilities to advance progress. In the future, researchers should not adhere to well-trodden paths only, avoid ideological blinkers, and expand their views, including modern experimental and integrative medicine approaches.

## Author contributions

WP invited experts from all the research fields, summarized the six published studies, and wrote part of the paper. SS summarized and supplemented the paper and wrote most of the content. GL made important supplements to the paper. All authors contributed to the article and approved the submitted version.
